# Neurodevelopmental Assessment in Children With Congenital Heart Disease by Applying the Denver Developmental Screening Test 2: A Prospective Cross-Sectional Study

**DOI:** 10.7759/cureus.33373

**Published:** 2023-01-04

**Authors:** Keta Vagha, Amar Taksande, Sneha Kenjale, Jayant Vagha, Ashish Varma

**Affiliations:** 1 Department of Pediatrics, Jawaharlal Nehru Medical College, Datta Meghe Institute of Medical Sciences, Wardha, IND

**Keywords:** neurodevelopmental assessment, acyanotic congenital heart disease, cyanotic congenital heart disease, pediatric population, denver developmental screening test, congenital heart diseases

## Abstract

Background

Congenital heart conditions often cause developmental delays and impact neurodevelopment throughout one’s lifetime. Hence, it is crucial to analyze the impact that heart defects have on the developing brain of a child. The present cross-sectional study was undertaken given the paucity of studies on the developmental status in children with congenital heart diseases (CHDs) in central India, where we tried to evaluate and compare the prevalence of neurodevelopmental delay in individuals with different congenital cardiac disorders. The objectives of our study were, firstly, to utilize the Denver Developmental Screening Test 2 (DDST-2) to evaluate the neurodevelopmental conditions in children with CHD; secondly, to compare the neurodevelopmental state of children with acyanotic CHD (ACHD) and cyanotic CHD (CCHD); and thirdly, to ascertain the prevalence of developmental delay in children with CHD.

Methodology

The study population comprised children aged six months to six years with two-dimensional (2D) echocardiography confirmation of CHD; those who were critically ill, had genetic syndromes, and were not willing to participate in the study were excluded. The neurodevelopmental assessment was conducted using the DDST-2. The screening looked at each patient’s progress in four areas: personal-social, fine motor-adaptive, language, and gross motor. Based on these observations, results were obtained and interpreted.

Result

Out of 82 children with CHD, the prevalence rate of developmental delay according to the DDST-2 was found to be maximum in the gross motor domain and the least affected in the social domain, which was similar to the analysis of developmental delay by developmental quotient (DQ). The comparative analysis of developmental delay in ACHD and CCHD according to the DDST-2 showed a significant P value only in the gross motor domain.

Conclusion

The DDST-2 is a straightforward screening tool for determining how well-developed infants with CHD are. The gross motor domain is the most frequently damaged in ACHD and CCHD, followed by the fine motor domain, and the social domain is the least affected. Cyanotic CHD patients are more susceptible to developmental delay than children with ACHD.

## Introduction

Congenital heart disease (CHD) is one of the most common and important causes of major congenital defects, generating major worldwide health problems. Heart defects are seen in 28% of all major congenital anomalies. As reported by various studies globally, the incidence of CHD ranges from four to 50 per 1,000 live births, whereas the prevalence is reported to be 9 per 1,000 live births [1]. With advances in the field of pediatric cardiology and cardiovascular thoracic surgery, about 85% of these children survive into adulthood [2]. Surviving children are at greater risk of developing neurodevelopmental delays because of various pathologies [3]. The impact is particularly severe in underdeveloped nations, where the majority of these births take place, due to high rates of morbidity and mortality. With improving survival, interest is increasingly focusing on the quality of life and long-term outcomes, particularly neurodevelopmental outcomes. Neurodevelopmental outcomes are a major concern for clinicians and parents. The frequency, range, and causes of neurodevelopmental outcomes associated with heart defects are still incompletely known. Unsurprisingly, neurodevelopmental challenges are common in children with congenital heart defects who also have an identifiable genetic condition or multiple congenital anomalies [4]. With the increasing complexity of CHD, the prevalence and severity of neurodevelopmental delays have increased with a greater incidence of CHDs with certain genetic syndromes [5]. According to recently released research, children with complex heart disorders are more likely to experience developmental delays in the areas of academic intelligence, expressiveness, receptivity, fine motor abilities, and gross motor skills [6]. The primary cause of this delay is hypoxia, which is more commonly documented in children with cyanotic congenital cardiac disorders [7]. In light of this, we made an effort to determine the prevalence of neurodevelopmental delay in CHD patients and compare the neurodevelopmental status between acyanotic CHD (ACHD) and cyanotic CHD (CCHD) using the Denver Developmental Screening Test 2 (DDST-2).

This article was previously presented as a meeting abstract at the Fourth Annual Summit on Infancy, Child Nutrition & Development 2018 on November 10, 2018, in Atlanta, USA.

## Materials and methods

**Figure 1:**
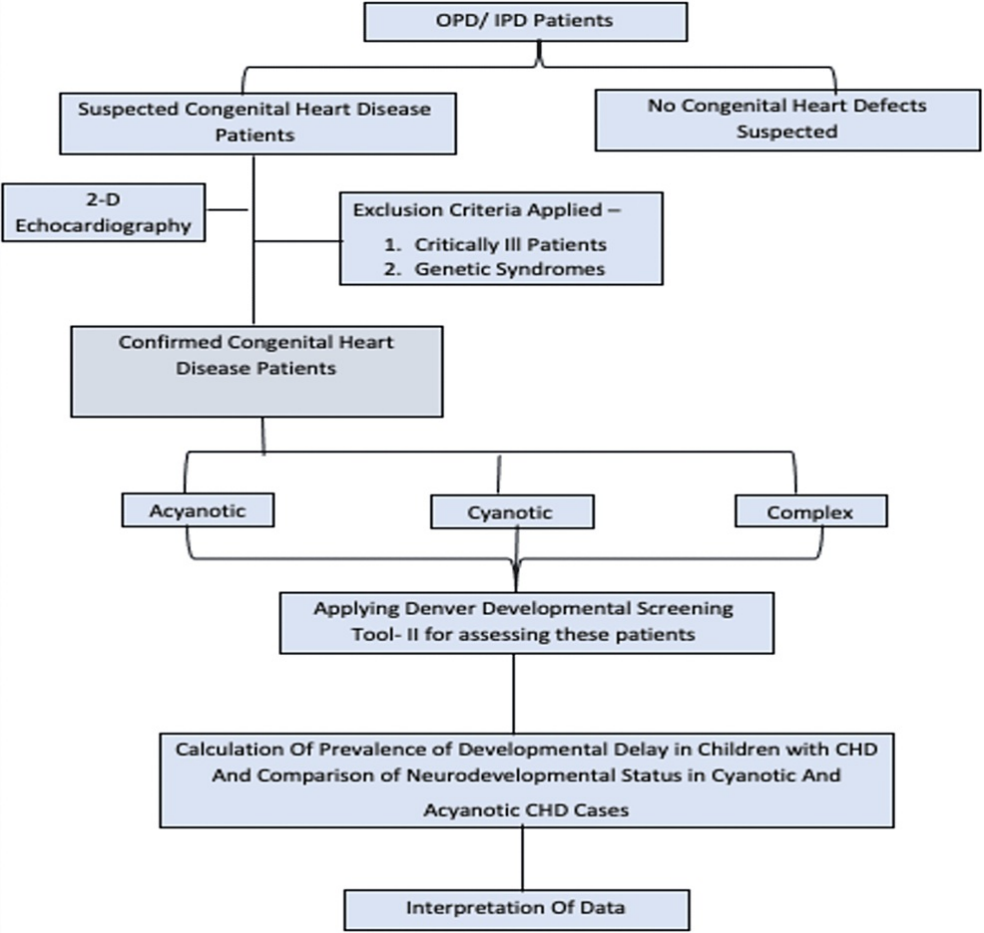
Flowchart showing the process of categorization OPD: outpatient department, IPD: inpatient department, 2D: two-dimensional, CHD: congenital heart disease

This prospective cross-sectional study was conducted in the Department of Pediatrics at Acharya Vinoba Bhave Rural Hospital, Sawangi (Meghe) located in Maharashtra, India, a 1,525-bed tertiary care hospital in Central India. The study was initiated after obtaining permission from the institutional ethics committee (DMIMS(DU)/IEC/2016-17/4088). All children aged six months to six years with suspected CHD on examination had undergone a two-dimensional (2D) echocardiography for confirmation. Children who were critically ill, had genetic syndromes, and were not willing to participate in the study were excluded. Based on the 2D echocardiography, details of the patient’s cardiac problem were recorded and then divided into either acyanotic, cyanotic, or complex heart diseases. The neurodevelopmental assessment was performed using the DDST-2 by screening each child for global development under four domains: personal-social, fine motor-adaptive, language, and gross motor. The history, anthropometric measures, systemic examination, type of CHD, interpretation of DDST-2 results, and developmental quotient (DQ) were noted in a predesigned proforma. Based on these observations, results were obtained and interpreted. The stratification procedure is depicted in the flowchart shown in Figure 1.

Statistical analysis

Microsoft Excel (Microsoft Corp., Redmond, WA, USA) was used to enter data. The Statistical Package for the Social Sciences (SPSS) version 17 (IBM SPSS Statistics, Armonk, NY, USA) for Windows was used to conduct all statistical analyses. Descriptive variables were presented as means, standard deviations (SDs), percentages, and averages. Different anthropometric and clinical parameters were compared between CHD patients with and without neurodevelopmental delay using the chi-squared test or Fisher’s exact test. Z score was developed using the National Center for Health Statistics (NCHS) data. P values of 0.05 or lower were considered significant. The expected prevalence of developmental delay in children with CHD was 30% with a precision of 90% and a confidence interval of 95%. Therefore, the required sample size was calculated to be 74 subjects. However, we conducted this study by enrolling 82 subjects, considering some percent of patient loss during the study.

## Results

**Figure 2:**
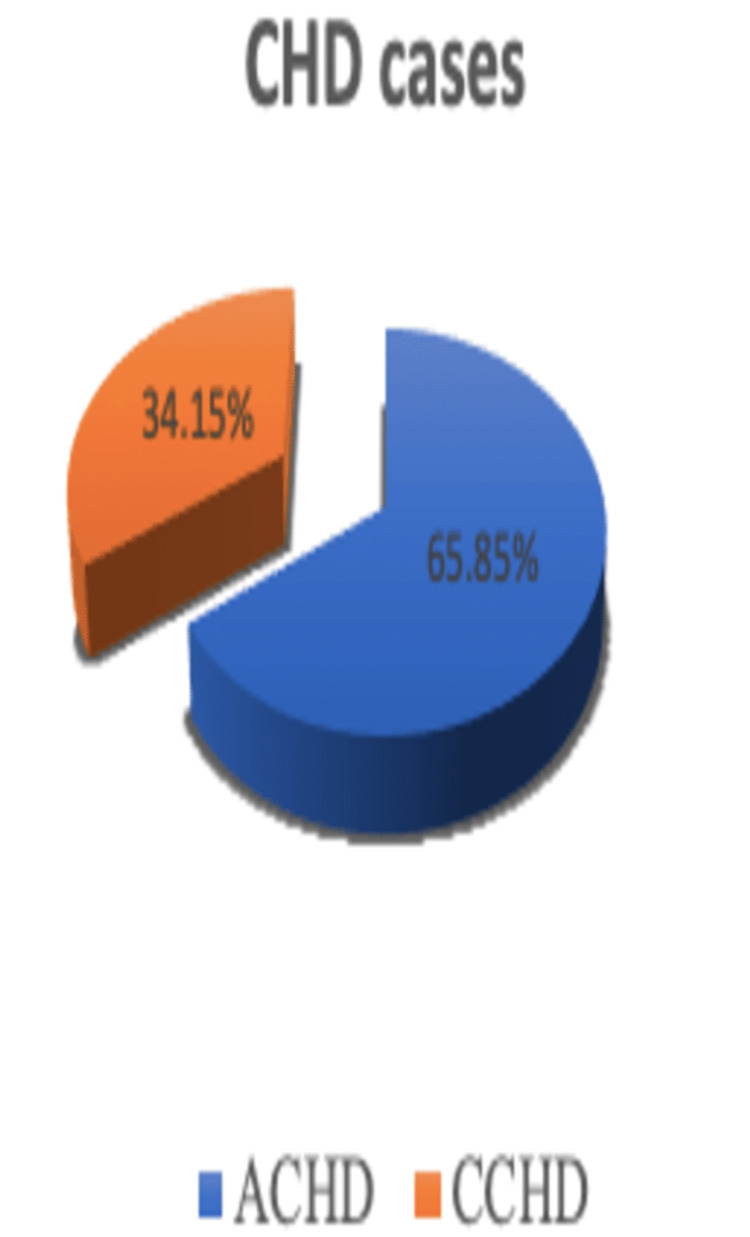
Pie chart denoting the number of congenital heart disease cases divided into ACHD and CCHD ACHD: acyanotic congenital heart disease, CCHD: cyanotic congenital heart disease

**Figure 3:**
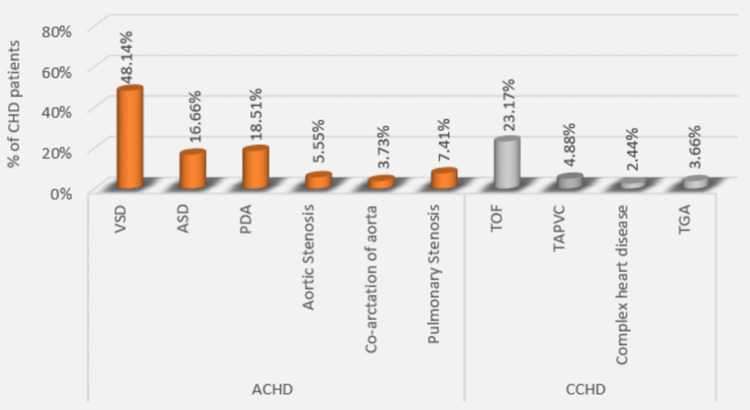
Bar graph depicting the various congenital heart diseases observed in this study VSD: ventricular septal defect, ASD: atrial septal defect, PDA: patent ductus arteriosus, TOF: tetralogy of Fallot, TAPVC: total anomalous pulmonary venous connection, TGA: transposition of great arteries

In this study, we have included data involving 82 patients diagnosed with CHD with no known neurological deficits. Out of 82 patients with CHD, 54 (65.85%) were of ACHD and 28 (34.15%) were of CCHD (Figure 2). Some other types of CHDs observed in our study are shown in Figure 3.

Children with varied age groups and gender were examined in this study, and their nutritional status was recorded (Table 1).

**Table 1:** Demographic and nutritional findings in congenital heart disease cases CHD: congenital heart disease, ACHD: acyanotic congenital heart disease, CCHD: cyanotic congenital heart disease, n: number of cases

Characteristics	Subcategories	CHD (n=82) (n (%))	ACHD (n=54) (n (%))	CCHD (n=28) (n (%))
Age	Below 1 year	12 (14.63)	3 (5.55)	9 (32.14)
	1-3 years	48 (58.53)	32 (59.25)	16 (57.14)
	4-6 years	22 (26.84)	19 (35.20)	3 (10.72)
Gender	Male	38 (46.34)	23 (42.55)	15 (53.55)
	Female	44 (53.66)	31 (57.45)	13 (46.45)
Wasting (weight for height)	Absent	54 (65.85)	35 (64.8)	19 (67.86)
	Present	28 (34.15)	19 (35.2)	9 (32.14)
Stunting (height for age)	Absent	57 (69.51)	38 (70.36)	19 (67.85)
	Present	25 (30.49)	16 (29.64)	9 (32.15)

Table 1 summarizes the age and gender distribution of patients with CHD and presents a comparative analysis of ACHD and CCHD. It also depicts an anthropometrical analysis of wasting and stunting in respective categories of CHD. In children under the age of five years, the World Health Organization (WHO) defines wasting as the weight for height that is 2 standard deviation (SD) below the median of the child growth standards and severe wasting as the weight for height that is 3 SD below the median [8]. Similarly, the WHO child growth standards for severe stunting is less than 3 SD from the median length/height for age [9], and stunting is less than 2 SD from the median length or height for age.

Of the four domains of development, the gross motor was relatively more affected, and the least affected domain was the social domain. Developmental quotient (DQ) below 70% was considered to be delayed. Table 2 shows the mean (±SD) developmental quotient in children with CHD.

**Table 2:** Developmental status in children with congenital heart disease (n=82) SD: standard deviation

Domain	Developmental quotient mean (±SD)
Gross motor	68.02 (±1.09)
Fine motor	70.20 (±0.88)
Language	69.81 (±0.98)
Social	73.5 (±1.20)

Out of 82 children with CHD, according to DQ, the maximum number of children had a delay in the gross motor domain, followed by a delay in the fine motor domain, and then in the language domain, and the least number of children had a delay in the social domain. Out of 54 children with ACHD, according to DQ, the maximum number of children had a delay in the gross motor domain, and the least was in the social domain. Similarly, out of 28 children with CCHD, according to DQ, the maximum number of children had a delay in the gross motor domain, and the least was in the social domain. It is observed that there is an increased delay in the CCHD group compared to the ACHD group. There is a high prevalence of gross motor domain delay in both types of CHDs, followed by the fine motor domain, and then the language domain, and the least affected was the social domain (Table 3).

**Table 3:** Developmental delay in children with congenital heart disease according to developmental quotient CHD: congenital heart disease, ACHD: acyanotic congenital heart disease, CCHD: cyanotic congenital heart disease, n: number of patients

Domain	CHD (n=82) (n (%))	ACHD (n=54) (n (%))	CCHD (n=28) (n (%))
Gross motor	54 (65.82)	31 (57.41)	23 (82.14)
Fine motor	52 (63.41)	31 (57.41)	21 (75.00)
Language	50 (60.98)	29 (53.70)	21 (75.00)
Social	32 (39.02)	19 (35.19)	13 (46.53)

Out of 82 children with CHD, according to the DDST-2, the maximum number of children had a delay in the gross motor domain and fine motor domain, followed by the language domain, and the least was in the social domain. Of the 54 children with ACHD, according to the DDST-2, the maximum number of children had a delay in the gross motor domain, followed by a delay in the fine motor domain and language domain, and the least was in the social domain. A similar trend was seen in 28 children with CCHD. According to the DDST-2, the maximum number of children had a delay in the gross motor domain, followed by a delay in the fine motor domain and then the language domain, and the least was in the social domain (Table 4). According to the DDST-2, more children with CCHD had developmental delay than children with ACHD, with an increased prevalence for the gross motor domain and the least delay in the social domain, which is similar to the analysis of developmental delay by DQ.

**Table 4:** Developmental delay in children with congenital heart disease according to the DDST-2 DDST-2: Denver Developmental Screening Test 2, CHD: congenital heart disease, ACHD: acyanotic congenital heart disease, CCHD: cyanotic congenital heart disease, n: number of patients

Domain	CHD (n=82) (n (%))	ACHD (n=54) (n (%))	CCHD (n=28) (n (%))
Gross motor	53 (64.53)	29 (53.70)	24 (85.71)
Fine motor	53 (64.53)	31 (57.41)	22 (78.57)
Language	52 (63.41)	31 (57.41)	21 (75.00)
Social	37 (45.12)	21 (38.89)	16 (57.14)

The comparative analysis of the number of children with developmental delay according to the DDST-2 in ACHD and CCHD cases showed a significant P value only in the gross motor domain and insignificant in all the other domains as shown in Table 5.

**Table 5:** Comparative developmental delay in acyanotic and cyanotic congenital heart disease cases according to the DDST-2 with significant P value only in the gross motor domain DDST-2: Denver Developmental Screening Test 2, ACHD: acyanotic congenital heart disease, CCHD: cyanotic congenital heart disease

Domain	ACHD (n=54) (n (%))	CCHD (n=28) (n (%))	P value
Gross motor	29 (53.70)	24 (85.71)	0.0045
Fine motor	31 (57.41)	22 (78.57)	0.0616
Language	31 (57.41)	21 (75.00)	0.1311
Social	21 (38.89)	16 (57.14)	0.1027

## Discussion

Congenital heart disease is invariably one of the leading causes of death in infancy. With quick identification and diagnosis, children with CHDs are now more likely to survive, and long-term neurodevelopmental outcomes are better because of rigorous clinical examination and innovative modalities [10]. Early screening for developmental delay can help reduce morbidity and help in the rehabilitation of these children [11]. In our hospital-based cross-sectional study, 82 children with CHDs were enrolled and screened for developmental delay by applying the DDST-2. Various studies have been done to establish the prevalence of neurodevelopmental delay in children with CHD who had different baseline clinical characteristics such as age, gender, and nutritional status.

In a study by Lata et al., the majority of children (62%) were between the ages of six and 12 months, while 29% were above 18 months [12]. In our present study of 82 children, the maximum number of children (n=48) were 1-3 years of age, followed by 22 children in the age group of 4-6 years and 12 children in the age group of <1 year. Children with CCHD had an earlier presentation because of a right to left shunt leading to their appearance of cyanosis, which was noticed by parents easily. In our study, the majority of cases were females (54%), and 46% were males, with a male/female ratio of 1:1.15. In the cases with ACHD, 57.4% were females and 42.5% were males, whereas in the cases with CCHD, 53.5% were males and 46.4% were females. On contrary, a study by Yilmaz et al. had a male/female ratio of 1.5:1; 61% were males and 39% were females [13]. In our study, 24% of the children with CHD had severe wasting and 31% had stunting. Of the 54 children that belonged to the ACHD group, 20% had severe wasting and 26% had severe stunting. The CCHD group consisted of 28 children, of which 32% had severe wasting and 43% had severe stunting. These findings demonstrate that children with CCHD are more predisposed to undernutrition than children with ACHD. Lata et al. showed weight <-3 SD was present in 66% of ACHD children and 37% of CCHD children; furthermore, regarding height, patients with a height <-3 SD was present in 19% of children with ACHD and 8% of children with CCHD [12].

A study by Polat et al. reported that out of 76 children with CHD, 11 patients had abnormal DDST-2 results, of which nine belonged to the hemodynamically impaired (HI) group and two belonged to the hemodynamically normal (HN) group, with a P value of <0.001 [14]. According to the composite score assigned to the failure points in every domain using the DDST-2, the HI group had a higher score in fine and gross motor domains as compared to the HN group, suggesting more of a delay in the HI group in assessing these domains. Another study conducted by Weinberg et al. found that out of 64 children, 64% developed normally according to the DDST-2, and the remaining were rated as abnormal (n=5), questionable (n=11), or untestable (n=7). It was observed that children with more severe CHD were significantly more likely to demonstrate an abnormal classification per the DDST-2 than children with no hemodynamic significance, which were significantly more likely to have a developmental delay per the DDST-2 than children with no hemodynamic significance [15].

Apart from the DDST-2 screening system, other studies with similar outcomes used different scoring systems. A total of 75 children with CHDs underwent the Japanese Denver Developmental Screening Test (JDDST), and Hirose et al. found that 18.7% of the newborns had a developmental delay in the gross motor domain and 4% in both the language and fine motor domains [16]. The Bayley Scale of Infant Development (BSID) is another screening system used to screen development outcomes. Yilmaz et al. studied neurodevelopment assessment in 38 children with CCHD using the BSID-2 [13]. When patients with isolated CCHD and patients with CCHD with other concomitant diseases were compared, the mean mental development index (MDI) and psychomotor development index (PDI) scores were lower in patients with CCHD with other concomitant diseases, but the difference was not statistically significant. According to the psychomotor development index, 41.6% of patients with isolated CCHD were determined to be mildly to severely retarded. Of the patients, 34.4% exhibited moderate or mild retardation based on the mental development index. Mussatto et al. found that by using the BSID-3 in children with CHD, developmental domains were strongly linked with one another. Of the individuals, 75% had results that fell into the “at risk” or “delayed” categories. A known genetic condition was present in 74% of affected children, such as 33% of those with single ventricle morphology, and 21% of those with double ventricular anatomy experienced significant developmental delay (cognitive, linguistic, or motor score of <70) [17].

Although the screening system was different in mentioned studies, the outcome in these cases was similar to ours. Some studies evaluated purely the motor domain in CHD patients. For instance, Leal et al. demonstrated the motor development scale in children with CHD [18]. The motor features assessed demonstrated a considerable risk of developing a motor delay (P=0.05), with the motor characteristics being significantly below the typical level. Motor development was observed to be impaired in 75% of the children with cyanotic heart disease and in 35.3% of children with ACHD by Lata et al. (P=0.001) when using the Developmental Assessment Scale for Indian Infants (DASII) [12]. In comparison to the acyanotic group, the cyanotic group’s mean DQ in the motor domain was considerably lower (P=0.048). ACHD and CCHD groups shared a similar mean mental DQ, with an insignificant P value of 0.92. In our study, out of 82 subjects, the developmental status of CHD as per the DQ for all four domains was as follows: gross motor, 68.02%; fine motor, 70.2%; language, 69.81%; and social, 73.5%. This shows that the gross motor domain was the most delayed domain and that the social domain is the least delayed domain. The mean DQ was lower in children with CCHD and mainly affecting the gross motor domain and least affecting the social domain. Out of 82 children with CHD according to the DQ, the maximum number of children with developmental delay was found in the gross motor domain, and the social domain had the minimum number of children with developmental delay. Similar results were found in the ACHD and CCHD groups. When the comparative analysis of developmental delay in ACHD and CCHD according to DQ was done, the P value was significant in the gross motor and language domains, whereas it was insignificant in the fine motor and social domains. A comparative analysis of the findings of these various studies along with our study has been summarized in Table 6.

**Table 6:** Comparative analysis of various studies that were mentioned DASII: Developmental Assessment Scale for Indian Infants, DQ: developmental quotient, ACHD: acyanotic congenital heart disease, CCHD: cyanotic congenital heart disease, DD: developmental delay, DDST-2: Denver Developmental Screening Test 2, CHD: congenital heart disease, BSID-3: Bayley Scale of Infant Development 3, JDDST: Japanese Denver Developmental Screening Test, MDI: mean mental development index, PDI: psychomotor development index

Author	Developmental screening tool	Conclusion
Lata et al. [12]	DASII DQ	CCHD had more DD than ACHD, and the motor domain was more affected.
Polat et al. [14]	DDST-2	Children with CHD with hemodynamic instability were more susceptible to DD than hemodynamically normal children.
Weinberg et al. [15]	DDST-2	Children with severe CHD had increased developmental delay.
Leal et al. [18]	Motor development scale	Delay in the motor domain in children with CHD.
Mussatto et al. [17]	BSID-3	Significant delay in children with syndromes and complex heart disease.
Hirose et al. [16]	JDDST	The majority had delays in the gross motor domain.
Yilmaz et al. [13]	BSID-2	Children with CCHD had lower PDI and MDI scores than normal children.
Our study	DDST-2	Children with CCHD were at more risk than children with ACHD. The gross motor domain is predominantly affected, followed by the fine motor domain, and the social domain is the least affected.

## Conclusions

In conclusion, there is a high prevalence of neurodevelopmental delay in children with CHD, and the DDST-2 is a simple screening tool to assess the developmental status of these children. Children with CCHD are more predisposed to developmental delay than children with ACHD, and the gross motor domain is the most affected in both ACHD and CCHD. Hence, early rehabilitation of developmentally delayed children with CHDs is essential for a better quality of life.
